# Hospital incident command system (HICS) performance in Iran; decision making during disasters

**DOI:** 10.1186/1757-7241-20-14

**Published:** 2012-02-06

**Authors:** Ahmadreza Djalali, Maaret Castren, Vahid Hosseinijenab, Mahmoud Khatib, Gunnar Ohlen, Lisa Kurland

**Affiliations:** 1 Karolinska Institutet, Department of Clinical Science and Education, Södersjukhuset, Stockholm, Sweden; 2 Karolinska Institutet, Department of Clinical Sciences and Education and Department of Emergency Medicine, Södersjukhuset, Stockholm, Sweden; 3 Department of emergency management, Natural Disaster Research Institute, Tehran, Iran; 4 Tehran social security organization, Tehran, Iran; 5 Karolinska Institutet, Department of Clinical Science, Intervention and Technology, Stockholm, Sweden

**Keywords:** Hospital Incident Command System, Performance, Exercise

## Abstract

**Background:**

Hospitals are cornerstones for health care in a community and must continue to function in the face of a disaster. The Hospital Incident Command System (HICS) is a method by which the hospital operates when an emergency is declared. Hospitals are often ill equipped to evaluate the strengths and vulnerabilities of their own management systems before the occurrence of an actual disaster. The main objective of this study was to measure the decision making performance according to HICS job actions sheets using tabletop exercises.

**Methods:**

This observational study was conducted between May 1st 2008 and August 31st 2009. Twenty three Iranian hospitals were included. A tabletop exercise was developed for each hospital which in turn was based on the highest probable risk. The job action sheets of the HICS were used as measurements of performance. Each indicator was considered as 1, 2 or 3 in accordance with the HICS. Fair performance was determined as < 40%; intermediate as 41-70%; high as 71-100% of the maximum score of 192. Descriptive statistics, T-test, and Univariate Analysis of Variance were used.

**Results:**

None of the participating hospitals had a hospital disaster management plan. The performance according to HICS was intermediate for 83% (n = 19) of the participating hospitals. No hospital had a high level of performance. The performance level for the individual sections was intermediate or fair, except for the logistic and finance sections which demonstrated a higher level of performance. The public hospitals had overall higher performances than university hospitals (P = 0.04).

**Conclusions:**

The decision making performance in the Iranian hospitals, as measured during table top exercises and using the indicators proposed by HICS was intermediate to poor. In addition, this study demonstrates that the HICS job action sheets can be used as a template for measuring the hospital response. Simulations can be used to assess preparedness, but the correlation with outcome remains to be studied.

## Background

Disasters, both natural and man-made, and the number of people affected by them have increased over the past decades [1,2]. The impact is illustrated by 46 million people being affected by earthquakes and tsunamis, between 1991 and 2005, worldwide [2]. Hospitals are cornerstones for health care in a community, and must continue to function in the face of a disaster [3,4]. An effective hospital command system is therefore crucial.

The Incident Command System (ICS) is a standardized on-scene, all-hazards incident management approach designed specifically to allow responders to adopt an integrated organizational structure equal to the complexity and demands of any single incident or multiple incidents without being hindered by jurisdictional boundaries [5,6]. The ICS was created in 1970 in response to a series of wildfires in Southern California in the United States [7,8]. The goal was to simplify communication and establish lines to authority and command [7,8], also, to provide more effective onsite utilization and management of resources [9]. The Incident Command System typically consists of five system functions as follows: Command, Planning, Operations, Logistics, and Financial/Administration [5,6].

The ICS was adapted in 1991 for use in the hospital based response to disasters, and duly renamed to the Hospital Emergency Incident Command System (HEICS) [10,11]. HEICS was revised in both 1992 and 1998. The most recent version, namely the Hospital Incident Command System (HICS), was presented in 2006 [12]. The HICS is currently the most commonly used model for hospital disaster response in the United States, also has been used in Taiwan and Turkey [13-18].

HEICS/HICS is a method by which the hospital operates when an emergency is declared [19]. It is the hospital's "standard operating procedure" whenever the hospital's disaster plan is activated, allowing the hospital to activate only those elements needed to address a specific emergency [19]. It also, using a common organizational terminology, facilitates communication between the hospital, first responders and other health-care facilities [19]. The overall objective is to facilitate the hospital response to disasters by superimposing a managerial structure for command and control, in addition to, a coordination of organizational missions e.g. administrative, logistical, informational, financial, and operational tasks [20].

The lack of internationally accepted standards of performance makes it difficult to evaluate the outcome of health care in a disaster [21]. Also, no generally accepted methodology exists for the evaluation of HICS or hospital-based exercises [21]. The evaluation of performance in disaster management is a topic of great interest [22,23]. Performance is a measure of how well an activity is done and can be measured in terms of accuracy, time, and quality [24,25]. The managerial activity for each position during the disaster response was determined in the relevant HICS job action sheets [12].

The vulnerability of Iran, with respect to natural disasters, especially earthquakes [26-28], necessitates an effective medical response to disasters. Iran's Ministry of Health and Medical Education required that all hospitals implemented the Hospital Incident Command System in 2007. Basic training courses in disaster medicine and the usage of HICS were developed by the medical science universities to support these efforts and were based on national guidelines [29].

The infrequent occurrence of major disasters is one reason that health care organizations remain ill equipped to systematically evaluate the strengths and vulnerabilities of their emergency management systems and programs [30]. Simulations are, therefore, important tools for the evaluation of disaster management and institutional performance [21,22,31-36]. A tabletop exercise is one type of simulation. It allows staff and key decision makers to discuss and act out an incident response under simulated emergency settings [3].

There has not previously, to our knowledge, been any previous study regarding the impact of the implementation of HICS on hospital performance with respect to response to a real or simulated disaster. The main objective of this study was to measure the decision making performance according to HICS job actions sheets using tabletop exercises.

## Methods

### Setting

This observational study was conducted in Iran, between May 1st 2008 and August 31st 2009. Hospitals that had implemented a hospital response system in accordance with the HICS and had a basic training course adapted to the HICS and were able to conduct a tabletop exercise were included. Twenty three hospitals were included in this study, due to financial constraints. None of these was excluded. Fourteen of the 23 hospitals were public and 9 were university hospitals. In Iran, there are three types of hospitals; university hospitals, private hospitals, and non-governmental (public) hospitals that belong to different organizations e.g. social security organization

Affiliation (public or university hospital), size (small: less than 100 beds; medium: 100-400 beds; large: more than 400 beds), use of a HICS advanced training course, the presence of a hospital command centre, and a hospital disaster plan, were assessed for each participating hospital.

### Table top exercises

A tabletop exercise was developed for each hospital. Tabletop exercises are simulated situations. Participants discuss the problems at hand, in depth, and make decisions regarding emergency responses, accordingly. One of the most important goals of a tabletop exercise is to compel the participant to make problem solving decisions. The decisions are documented to serve as a reference with which to evaluate the exercise [3,36,37].

Each exercise was based on a risk map of the area. The most likely hazard for each given hospital was chosen as the disaster scenario for the exercise.

Each scenario was run for a maximum of 2 hours. There are 28 standardized scenarios in the HICS-2006 including earthquake, fire and chemical emergencies. The content of these scenarios was extended with respect to geographical information, characteristics of the disaster, and process of influx of disaster casualties. A facilitator, one of the evaluators, initiated the discussion and directed the participants toward in-depth problem solving. All positions of the HICS were assessed according to the 5 main sections (command, operations, planning, logistics, and finance/administration) using the job action sheets, during both the exercise and the evaluation process [8]. The participants were asked to document all decisions made during the exercise. The number of participants differed due to the size of the participating hospitals. There was one person for each position at 4 hospitals, but in 19 hospitals some personnel was responsible for two positions, e.g. a person for "Medical Gases" and "Medical Devices".

The scoring according to the job action sheets was done by a group of three evaluators. The evaluators were medical doctors, who had worked in the field of HICS and HDP, for at least 6 years. They had international experiences from training programs and visits of hospital using HICS. The same group of evaluators was used to for all 23 exercises. There was a team discussion after each exercise. The about scoring of HICS performance by the evaluators was completed in consensus.

### Measurement of performance

The HICS job action sheets were used as the performance indicators for decision making (see additional file 1) [8]. The job action sheet is an incident management tool designed to familiarize the user with critical aspects of the command position he or she is assuming. It includes title, purpose, to whom they report, and critical action considerations. These tasks are intended to prompt the incident management team members to take needed actions related to their roles and responsibilities [12]. The performance for each HICS position was scored based on the compatibility of the participants' decisions with the relevant job action sheet. A compatibility below 40% was scored as 1, a compatibility between 40 and 70% as 2, and if the compatibility was above 70%, the score was 3. The score was 0 if the position was missed or no performance was achieved for the specific task.

The range for the total HICS score was 1-192 (see additional file 1), and was divided into at three categories: Fair: 1-76; Intermediate: 77-134; High: 135-192. These cut-off values are based on expert consensus.

The evaluators compared the participants' documented decisions with the content according to the job action sheets, as a measure of compatibility. The evaluators agreed on to what extent the duties in a job action sheet are provided by the documented decisions of the participant (HICS member).

### Statistical analysis

Descriptive statistics was performed. Measures of central tendency were used for HICS and its main sections performance scores. The HICS scores were normally distributed except for the scores for the planning section, as tested by a normal probability plot [38]. A t-test was used to compare means of HICS performance between hospitals with respect to their affiliation, size and training courses. A Univariate Analysis of Variance was used to evaluate the effect of independent variables on HICS performance. A p-value of less than 0.05, two tailed, was considered to be significant.

The SPSS 17 (IBM, New York, USA) was used for data analysis.

## Results

The public hospitals had implemented the HICS earlier than the university (community) hospitals; and had also been provided with a budget with which to conduct relevant training programs.

Background information for the participating hospitals is presented in Table 1. The hospitals were located in 12 different cities including Tehran and 1-2 hospitals from each disaster regional collaborating center of Iran's Ministry of Health. Their location is not disclosed in detail due to confidentiality reasons, in accordance with WHO's recommendations. At the time of this study, there were 9 regional disaster centers of Iran's Ministry of Health. None of the participating hospitals had a hospital disaster plan. Only two hospitals had a designated Hospital Command Centre. The exercise included one of the following hazards; earthquake (n = 15), accident with hazardous materials (n = 5), and fire (n = 3).

**Table 1:** Background for the 23 hospitals that participated in the tabletop exercises and HICS performance

History of advanced HICS training course* n (%)	No 8 (35%) Yes 15 (65%)
**Hospital Disaster Plan n (%)**	Present 0 (0%)

**Hospital Command Centre n (%)**	No 21 (91%) Yes 2 (9%)

Hospital size** n (%)	Small 6 (26%) Medium 17 (74%)

*HICS training modules

**Small: less than 100 beds, Medium:100-400 beds

The lowest total HICS score was 56 and the highest was 119, with a mean of 85 (± 15 SD). The logistic and financial/administration sections received the highest scores, and the planning section had the lowest score (Table 2).

**Table 2:** HICS score presented for each section for the 23 participating hospitals.

Sections of HICS	Mean ± SD	Range
Command	5.2 ± 1.7	3-9

Operations	43.1 ± 7.8	26-58

Planning	8.6 ± 2.9	5-17

Logistics	19.1 ± 3.7	12-26

Financial/Administration	9.0 ± 1.8	6-13

Total HICS	85.0 ± 15.6	56-119

**Figure 1:**
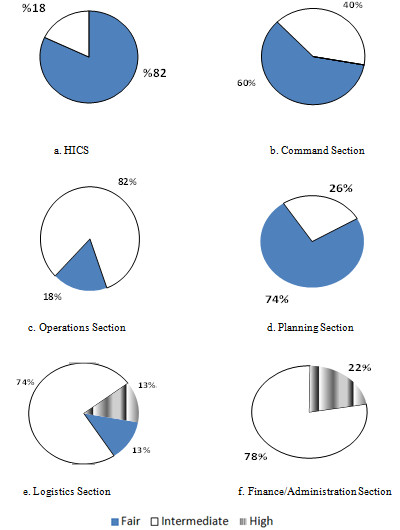
**Performance level as presented in pie charts for a. the total HICS score (a sum of b-f), b. Command Section score, c. Operations Section score, d. Planning Section score, e. Logistics Section score, f. Finance/Administration Section score**.

HICS performance was intermediate for 19 hospitals (83%). No hospital had a high level of performance. The performance level of the different individual sections was fair to intermediate, except for the logistics and finance/administration sections which had a high level of performance in 3 and 5 hospitals, respectively (Figure 1).

The HICS and its main sections in the public hospitals had a higher performance than university hospitals (Table 3), except for the operations section where there was no significant difference in performance (P = 0.07).

**Table 3:** Results of the tabletop exercise presented as the HICS score for each section for the 23 participating hospitals in relation to their affiliation, ie.

Sections of HICS	Mean ± SD		*p*-value (2-tailed)
	
	University (n = 9)	Public (n = 14)	
Command	4.1 ± 0.8	5.9 ± 1.7	0.008

Operations	39.4 ± 8.8	45.5 ± 6.4	0.07

Planning	7.1 ± 0.8	9.5 ± 3.2	0.04

Logistics	16.7 ± 2.3	20.6 ± 3.7	0.009

Financial/Administration	7.7 ± 0.7	9.9 ± 1.8	0.001

Total HICS	75.0 ± 11.9	91.4 ± 14.5	0.01

whether they are public or university hospitals.

Hospitals with both basic and advanced courses had a higher level of performance (89.2 ± 15.5) as compared to hospitals with only the basic course (77.1 ± 13.3), although the difference was not significant (P = 0.07).

There was no significant difference between small and medium hospitals (P = 0.09). No large hospitals participated in the study.

Public affiliation was the only independent predictor of the HICS performance (P = 0.04) in a regression model including medium size, public affiliation, advanced course, and earthquake scenario.

## Discussion

This study showed that the decision making performance, as measured by the indicators proposed by the Hospital Incident Command System (HICS), during tabletop exercises was intermediate for the majority of the participating hospitals and poor in a few, while none showed a high performance. Previous studies evaluating hospital management during simulated disasters demonstrated a similar level of performance [23,39-41]. However, these studies [23,39-41] have only evaluated decision making with respect to the command and control functions, and not the overall organisation. It is necessary to evaluate all components of the hospital disaster response in order to assess the efficacy of the hospital response to a disaster. We believe that one way to accomplish this is by using the HICS job action sheets as a template for measuring performance.

The performance at public hospitals was significantly higher than that of university hospitals. There are, to our knowledge, no previous studies on performance as measured by HICS and hospital affiliation. However public hospitals did not appear to be superior to other hospitals, with respect to disaster preparedness as measured by practice variation, plan characteristics, and surge capacity in a previous study [42,43].

HICS was implemented at public hospitals approximately one year before that of university hospitals. In addition, there is more financial support for training courses and drills at public hospitals as compared to university hospitals. Thus, public hospitals have a longer experience of HICS and are also better funded, which may explain our results. Previous studies demonstrated that funding, standards, and experience in disaster management are the improving factors for hospital disaster preparedness [4].

Training courses had no significant impact on HICS performance according to the current study. Also a review from 2004 showed no support for training courses influencing performance [44]. More recent studies have, however, shown that disaster management training courses enhance individual knowledge and skills [29,45]. The content, construction and execution of the training courses were beyond the scope of this study, which may, in part, influence our results. Interventional studies including standardized training courses are needed.

The hospital size did not affect the performance as measured by HICS according to our results. There are, however, previous studies suggesting that size is an important function of hospital capacity in disaster response [46-48]. However, there is no consensus on size in relation to preparedness [43]. There are no previous reports regarding hospital size and managerial performance as measured by HICS. We believe that hospital performance is an effect of preparedness and not size per se.

HICS was implemented in Iran in 2007. Therefore, one may infer that the lack of high performance may be explained by an insufficient understanding of HICS and also an incompatibility of HICS with the pre-existing management structure. However, we believe that the intermediate to low performance is a consequence of the lack of a comprehensive hospital disaster plan and a hospital command centre (HCC). There are, however, to date, no studies on the effect of implementing a disaster plan on HICS performance.

### Limitations

A limitation of this study is that it is performed in Iran which restricts the generalizability of our results. However, the HICS and its job action sheets are standardized and used worldwide. Despite the lack of internationally accepted standards of performance for disaster health management [21,30] the HICS does have international recognition.

An additional limitation is that we have not measured inter-rater reliability. We did, however, have a team discussion after each exercise and the evaluators independently came to the same HICS score. Another limitation is that the cut-off levels for the performance levels are arbitrary. However, they were based on expert consensus. Standardizing these cut-off levels requires prospective outcome based studies.

Additionally, there were too few participating hospitals to allow for the testing the impact of different scenarios for the same hospital. Only one scenario was used for each hospital, which limits the results to these hazards. Other hazards would be of interest, such as hazardous materials, fire and power shortage. However, the hazard chosen for each hospital was that of highest risk.

Using tabletop exercises may of course be questioned with respect to the validity of assessing hospital preparedness [31-36]. However, decision making performance, as measured in tabletop exercises, has impact on preparedness as measured in other functional exercises [41]. Outcome studies remain to be performed.

## Conclusions

The decision making performance in the participating Iranian hospitals, as measured during tabletop exercises and using the Hospital Incident Command System (HICS), was intermediate to poor. The performance was better in the public hospitals as compared to university hospitals and was shown to be independent of the hospital size.

The HICS job action sheets can be used as a template for measuring the hospital response. We believe that a comprehensive hospital disaster plan should include not only managerial and operational elements of hospital preparedness, but also an appropriate command system suited to the specific hospital organisation.

Simulations can be used to assess preparedness, but the correlation with outcome remains to be studied.

## List of Abbreviations

HICS: Hospital Incident Command System

## Competing interests

The authors declare that they have no competing interests.

## Authors' contributions

ARD was involved in the study design, data collection, analysis, and manuscript writing. MC contributed to the analysis of the data and to the writing of the manuscript. VH and MK were involved in the study design and took an active part in the data collection and the interpretation of the results. GO participated in the study design, writing-up and finalization of the manuscript. LK participated in the study design, analysis and the manuscript writing, revision and editing. All authors read and approved the final manuscript.

## Supplementary Material

Additional file 1 **Indicators of decision making performance of Hospital Incident Command System in five different sections; achieved results on each indicator given 0, 1, 2, or 3 points**. Click here for file
