# Supplementary material for: Hospital incident command system (HICS) performance in Iran; decision making during disasters
Source: Scand J Trauma Resusc Emerg Med. 2012 Feb 6;20:14. doi: 10.1186/1757-7241-20-14 (PMC3296571; doi:10.1186/1757-7241-20-14)
Supplement: Additional file 1 — Indicators of decision making performance of Hospital Incident Command System in five different sections; achieved results on each indicator given 0, 1, 2, or 3 points. [file 1757-7241-20-14-S1.DOC]

**Additional file 1- Indicators of decision making performance of Hospital Incident Command System in five different sections; achieved results on each indicator given 0, 1, 2, or 3 points**

| **Section** | **Indicators of decision making performance** | **Score** |
| --- | --- | --- |
| **Command group** | Organize and direct the Hospital Command Center (HCC). Give overall strategic direction for hospital incident management and support activities. | 1-15 |
|  | Serve as the conduit for information to internal and external stakeholders, the news media, visitors, etc as approved by the Incident Commander. |  |
|  | Ensure safety of staff, patients, and visitors, monitor and correct hazardous conditions. To halt any operation that poses immediate threat to life and health. |  |
|  | Function as the incident contact person in the Hospital Command Center for representatives from other agencies. |  |
|  | Maintain hospital’s capability and services. Advice the Incident Commander or Operations Section Chief on issues related to hospital operations. |  |
| **Operations section ^٭^** | Develop and implement strategy and tactics to carry out the objectives established by the Incident Commander. | 0-99 |
|  | Organize and manage the deployment of supplementary resources. |  |
|  | Organize and manage the deployment of personnel resources. |  |
|  | Organize and manage the deployment of vehicle resources. |  |
|  | Organize and manage the deployment of equipment and supplies. |  |
|  | Organize and manage the deployment of medication. |  |
|  | Organize and manage the delivery of emergency, inpatient, outpatient, and casualty care, and clinical support services. |  |
|  | Assure treatment of inpatients, manage the inpatient care area(s), and provide for a controlled patient discharge. |  |
|  | Prepare outpatient service areas to meet the needs of in-house and newly admitted patients. |  |
|  | Assure delivery of emergency care to arriving patients. |  |
|  | Address issues related to mental health emergency response, manage the mental health care area, and coordinate mental health response activities. |  |
|  | Organize and manage clinical support services. Assist in providing the optimal functioning of these services. Monitor the use and conservation of them. |  |
|  | Coordinate inpatient and outpatient registration. |  |
|  | Organize and manage the services required to sustain and repair the hospital’s infrastructure operations. |  |
|  | Maintain power and lighting to the hospital and campus facilities. Ensure adequate generator fuel. |  |
|  | Evaluate and monitor the patency of existing water, sewage, and sanitation systems. Enact pre-established alternate methods of waste disposal. |  |
|  | Maintain heating and air conditioning to the facility and adjacent facilities. |  |
|  | Organize and manage the services required to sustain and repair the hospital’s buildings and grounds. |  |
|  | Organize and distribute medical gases to requesting clinical care areas. |  |
|  | Organize and distribute medical devices to requesting clinical care areas. |  |
|  | Ensure proper cleaning and disinfection of hospital environment. |  |
|  | Organize and maintain food preparation and delivery services. |  |
|  | Organize and direct hazardous material incident response activities. |  |
|  | Coordinate detection and monitoring activities related to hazardous material. |  |
|  | Coordinate the response activities related to hazardous material spill. |  |
|  | Coordinate the patient decontamination activities. |  |
|  | Coordinate facility and equipment decontamination activities. |  |
|  | Coordinate all of the activities related to personnel and facility security. |  |
|  | Ensure the security by monitoring individuals entering and exiting the building. |  |
|  | Maintain scene safety and ensure crowd control. |  |
|  | Organize and enforce vehicular traffic security for facility. |  |
|  | Coordinate the search and rescue of missing staff, patients and family members. |  |
|  | Coordinate security of facility with outside law enforcement agencies. |  |
| **Planning section** | Oversee all incident-related data gathering and analysis regarding incident operations and assigned resources, develop alternatives for tactical operations, conduct planning meetings and prepare the Incident Action Plan for each period. | 0-27 |
|  | Maintain information on the status, location, and availability of personnel, teams, facilities, supplies, and major equipment to ensure availability of use during the incident. Maintain a master list of all resources assigned to incident operations. |  |
|  | Maintain information on the status, location, and availability of on-duty staff and volunteer personnel. |  |
|  | Maintain information on the status, location, and availability of equipment and supplies in the inventory and additional materiel received from outside agencies. |  |
|  | Collect, process, and organize ongoing situation information; prepare situation summaries; and develop projections and forecasts of future events related to the incident. Prepare maps and gather and disseminate information and intelligence for use in the Incident Action Plan. |  |
|  | Monitor and document the location of patients at all times within the hospital's patient care system, and track the destination of all patients departing the facility. |  |
|  | Maintain information on the status, location, and availability of all patient beds, including disaster cots and stretchers. |  |
|  | Maintain accurate and complete incident files, including a record of the hospital’s response and recovery actions and decisions; provide duplication services to incident personnel; and file, maintain, and store incident files for legal, analytical, and historical purposes. |  |
|  | Develop and coordinate an Incident Demobilization Plan that includes specific instructions for all staff and resources that will require demobilization. |  |
| **Logistics section** | Organize and direct those operations associated with maintenance of the physical environment and with the provision of human resources, materiel, and services to support the incident activities. | 0-36 |
|  | Organize and manage the services required to maintain the hospital’s communication system, food and water supply for staff, and information technology and systems. |  |
|  | Organize and coordinate internal and external communications connectivity. |  |
|  | Provide computer hardware, software and infrastructure support to staff. |  |
|  | Organize food and water stores and prepare for rationing during periods of anticipated or actual shortage. |  |
|  | Organize and manage the services required to maintain the hospital’s supplies, facilities, transportation, and labor pool. Ensure the provision of logistical, psychological, and medical support of hospital staff and their dependents. |  |
|  | Ensure the availability of medical care for injured or ill staff. Ensure the availability of behavioural and psychological support services to meet staff needs during and following an incident. Coordinate prophylaxis/vaccination/ immunization of staff. Coordinate medical surveillance program for employees. |  |
|  | Ensure the availability of medical, logistic and mental health and day care for the families of staff members. Coordinate prophylaxis/ vaccination/ immunization of family members. |  |
|  | Acquire, inventory, maintain, and provide medical and non-medical care equipment, supplies, and pharmaceuticals. |  |
|  | Organize, manage and support building systems, equipment and supplies. Ensure proper cleaning and disinfection of hospital environment. |  |
|  | Organize and coordinate the transportation of all ambulatory and non-ambulatory patients. Arrange for the transportation of human and material resources within or outside the facility. |  |
|  | Collect and inventory available staff and volunteers at a central point for assignment by the Staging Officer. Maintain adequate numbers of both medical and non-medical personnel. Assist in the maintenance of staff morale. |  |
| **Finance/ Administration section** | Monitor the utilization of financial assets and the accounting for financial expenditures. Supervise the documentation of expenditures and cost reimbursement activities. | 0-15 |
|  | Documentation of personnel time records. Monitor and report on regular and overtime hours worked/volunteered. |  |
|  | Administering accounts receivable and payable to contract and no contract vendors. |  |
|  | Receiving, investigating and documenting all claims reported to the hospital during the incident, which is alleged to be the result of an accident or action on hospital property. |  |
|  | Providing cost analysis data for the declared emergency incident and maintenance of accurate records of incident cost. |  |
| Total HICS |  | 1-192 |

**^٭^** The business continuity branch wasn’t included in national guideline for Iran’s hospitals; therefore we exclud
